# Copy Number Variant Detection by NIPT: Biological Constraints and the Limits of Prenatal Genomic Inference

**DOI:** 10.3390/genes17060636

**Published:** 2026-05-30

**Authors:** Dorina Merhala, Béla Veszprémi, Réka Anna Vass

**Affiliations:** 1 Doctoral School of Health Sciences, University of Pécs, 7622 Pécs, Hungary; merhala.dorina@czeizelintezet.hu (D.M.); veszpremi.bela@pte.hu (B.V.); 2 Department of Obstetrics and Gynecology, Medical School, University of Pécs, 7622 Pécs, Hungary

**Keywords:** copy number variants, cell-free fetal DNA, non-invasive prenatal testing, confined placental mosaicism, prenatal genomic inference

## Abstract

Background: Non-invasive prenatal testing (NIPT) based on analysis of Cell-Free Fetal DNA has transformed screening for common aneuploidies and is increasingly extended to genome-wide detection of copy number variants (CNVs). However, CNV detection remains constrained by analytical limitations and biological signal complexity. Methods: This review evaluates the analytical validity, biological constraints, and clinical interpretation challenges of CNV detection by NIPT, framing it as a probabilistic genomic inference rather than a direct measure of fetal copy number. Results: Performance depends on sequencing depth, bin resolution, fetal fraction, guanine–cytosine correction, and reference modeling, leading to variable detection thresholds. The predominantly placental origin of cfDNA introduces discordance through Confined Placental Mosaicism, post-zygotic events, and clonal variation. Maternal CNVs, mosaicism, vanishing twin, and occult malignancy further complicate interpretation and may cause false positives. Clinical validity is heterogeneous, with positive predictive value dependent on CNV size, genomic context, and prevalence. Reporting practices remain inconsistent. Conclusions: CNV detection by NIPT is fundamentally limited by interpretation of a composite maternal–placental signal. Progress requires improved tissue-of-origin discrimination, multi-omic integration, and standardized reporting to ensure responsible clinical implementation.

## 1. Introduction

Non-invasive prenatal testing (NIPT) based on analysis of circulating cell-free DNA (cfDNA) in maternal plasma has transformed prenatal screening since its clinical introduction in 2011. The discovery that fetal-derived cfDNA fragments are detectable in maternal circulation [1] enabled the development of massively parallel sequencing approaches capable of identifying common autosomal trisomies with high sensitivity and specificity [2,3]. Professional societies subsequently endorsed NIPT as a primary or contingent screening tool for trisomy 21, 18, and 13 in both high- and average-risk populations [4,5]. As sequencing costs have declined and bioinformatic pipelines have matured, commercial platforms have progressively expanded testing beyond whole-chromosome aneuploidies toward genome-wide detection of subchromosomal copy number variants (CNVs). CNVs contribute substantially to genomic disorders associated with developmental delay and congenital anomalies, and chromosomal microarray analysis (CMA) has become the diagnostic standard in pregnancies with structural abnormalities [6,7]. The possibility of detecting clinically significant CNVs non-invasively has therefore generated considerable interest. However, unlike invasive diagnostic testing, NIPT analyzes fragmented cfDNA derived predominantly from placental trophoblasts rather than directly from fetal tissues [1,8]. This biological origin introduces inherent limitations, including confined placental mosaicism (CPM), which may lead to discordant results between cfDNA screening and fetal karyotype [9,10]. Post-zygotic mitotic errors, variable mosaic fractions, and differential placental–fetal lineage allocation further complicate interpretation of subchromosomal imbalances detected in maternal plasma.

From an analytical perspective, genome-wide CNV detection by NIPT relies on shallow whole-genome sequencing with statistical comparison of read-depth distributions across genomic bins. Detection sensitivity is influenced by sequencing depth, bin size, GC content correction, fetal fraction, and reference normalization models [11,12]. Consequently, resolution thresholds are probabilistic and platform-dependent, and the minimum detectable CNV size varies considerably across laboratories. Importantly, positive predictive value (PPV) is strongly dependent on CNV size and underlying population prevalence, raising concerns about clinical validity in low-risk populations [13].

Additional biological confounders further challenge interpretation. Maternal CNVs, low-level maternal mosaicism, vanishing twin phenomena, and, in rare cases, occult maternal malignancy can generate abnormal cfDNA signals unrelated to the fetal genome [14,15,16]. These factors complicate counseling and underscore the distinction between screening and diagnostic testing. Despite rapid commercial expansion of genome-wide NIPT panels, reporting thresholds, handling of mosaic findings, and disclosure of variants of uncertain significance remain inconsistently standardized.

In this review, we critically evaluate the analytical validity, biological constraints, and clinical interpretation challenges associated with CNV detection by NIPT. By integrating technical, biological, and clinical considerations, we aim to clarify the evidentiary limits of current approaches and highlight priorities for harmonized validation and responsible implementation.

From a clinical perspective, genome-wide CNV detection by NIPT should be regarded as an extension of screening rather than a substitute for diagnostic genomic testing. In pregnancies with structural anomalies or high clinical suspicion of chromosomal imbalance, invasive testing with chromosomal microarray analysis remains the recommended standard. While expanded NIPT panels may increase detection of subchromosomal abnormalities, their use in low-risk populations requires careful consideration of reduced positive predictive value and increased potential for false-positive or uncertain findings.

## 2. Analytical Validity of CNV Detection

The analytical validity of CNV detection by NIPT depends on the interplay between sequencing technology, bioinformatic modeling, and biological signal composition. Unlike diagnostic chromosomal microarray analysis performed on fetal tissue, cfDNA-based CNV detection relies on statistical inference from fragmented, mixed maternal–placental DNA present at low fractional abundance [1,10]. As such, analytical resolution is not fixed but probabilistic and highly context-dependent. The principal sequencing and analytical approaches currently used for CNV detection by NIPT differ substantially in genomic coverage, analytical resolution, and clinical applicability. A comparative overview is summarized in Table 1.

### 2.1. Shallow Whole-Genome Sequencing (sWGS)

Shallow whole-genome sequencing (typically 0.1–0.5× coverage) is the most widely used approach for genome-wide CNV detection in NIPT [11,17]. In this method, millions of short cfDNA fragments are sequenced and mapped across the genome, and read counts are aggregated into predefined genomic bins. Deviations from expected representation are assessed relative to reference datasets using statistical normalization models [12]. Because coverage is low, CNV detection relies on subtle shifts in read-depth distributions rather than direct breakpoint identification. Analytical sensitivity declines as CNV size decreases, fetal fraction drops, or sequencing depth is reduced [18]. Smaller CNVs generate proportionally smaller deviations in read representation, making discrimination from background noise increasingly challenging.

### 2.2. Targeted CNV Panels

Targeted approaches enrich specific genomic regions associated with recurrent microdeletion or microduplication syndromes (e.g., 22q11.2 deletion syndrome). By concentrating sequencing reads within selected loci, targeted panels can achieve higher effective depth per region compared with genome-wide shallow sequencing [19,20]. This enrichment may improve sensitivity for small recurrent CNVs. However, targeted panels sacrifice genome-wide coverage and may be vulnerable to locus-specific amplification bias. Moreover, validation studies frequently rely on enriched or high-risk cohorts, limiting extrapolation to population screening settings [20].

### 2.3. Read Depth Requirements

Sequencing depth directly influences signal-to-noise ratio. Increased read counts reduce stochastic variation across genomic bins, thereby improving detection of smaller CNVs [11,12]. Nevertheless, increasing depth cannot fully compensate for low fetal fraction, as signal amplitude remains biologically constrained by the fractional representation of placental DNA in maternal plasma [1,18]. Many clinical platforms operate at depths optimized for whole-chromosome aneuploidy detection; subchromosomal CNV detection is often implemented within this framework rather than through assays independently optimized for higher resolution [17].

### 2.4. Bin Size and Resolution Limits

Genomic bins typically range from approximately 50 kb to 1 Mb [11,12]. Smaller bins increase theoretical resolution but amplify variance, whereas larger bins reduce variance at the expense of breakpoint precision. Consequently, minimum detectable CNV size is influenced by bin configuration, normalization strategy, and statistical thresholds. Reported detection thresholds (commonly 5–10 Mb for genome-wide CNVs) reflect empirical validation parameters rather than intrinsic biological boundaries [17,18].

### 2.5. Relationship Between Fetal Fraction and Minimum Detectable CNV Size

Fetal fraction is a central determinant of analytical performance. Because cfDNA in maternal plasma is a mixture of predominantly maternal and placental DNA, the fractional representation of a fetal CNV is diluted by maternal background [1,9]. For a heterozygous fetal deletion, the expected proportional shift in read depth approximates half the fetal fraction. Thus, at a fetal fraction of 10%, the anticipated deviation in representation is approximately 5%, and proportionally lower at reduced fetal fractions. As fetal fraction decreases, larger CNVs are required to produce statistically distinguishable signals above background noise [9,18]. Conversely, higher fetal fractions improve detectability, assuming sufficient sequencing depth and appropriate normalization. Therefore, minimum detectable CNV size is dynamically dependent on fetal fraction rather than representing a fixed technical threshold.

### 2.6. GC Bias Correction

GC content strongly influences amplification efficiency and sequencing representation. Regions of extreme GC content may be over- or under-represented, generating systematic biases that can mimic or obscure copy number changes [11,12]. Correction algorithms—commonly based on LOESS regression or reference-based normalization—adjust read counts according to GC content to mitigate these effects [12]. However, incomplete correction may leave residual artifacts, particularly in GC-rich or segmentally duplicated regions, limiting reliability in specific genomic contexts.

### 2.7. Noise Modeling

Background noise arises from technical variation (library preparation, sequencing chemistry, mapping artifacts) and biological variability (fragmentation patterns, maternal mosaicism) [10,18]. Statistical frameworks typically model expected read distributions using z-score methodologies, normalized chromosome values, or hidden Markov models [11,12]. Significance thresholds are defined relative to reference populations. Importantly, noise characteristics vary across laboratories and sequencing platforms, meaning that identical CNVs may be detectable in one analytical setting yet fall below reporting thresholds in another [17]. Validation datasets, reference cohort composition, and bioinformatic parameters therefore materially influence reported sensitivity and specificity.

### 2.8. Key Analytical Concept: Resolution Is Probabilistic, Not Absolute

**Figure 1:**
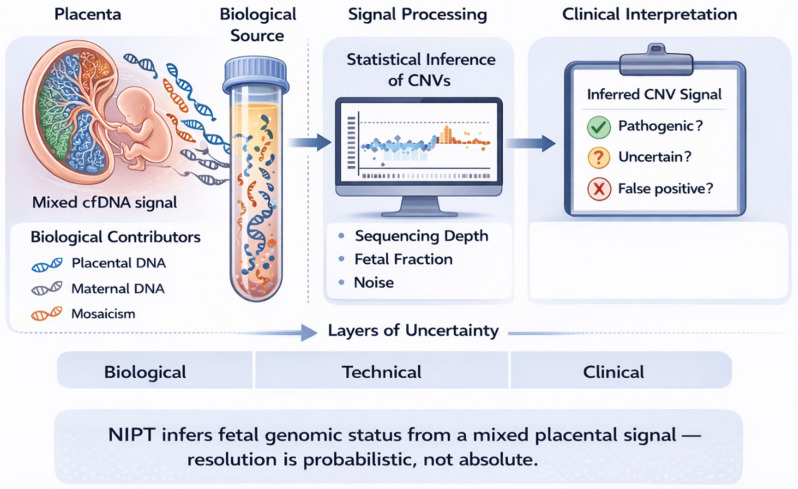
Conceptual model of the CNV-NIPT pathway and sources of uncertainty.

Unlike chromosomal microarray or fetal genome sequencing, CNV detection by NIPT does not directly measure fetal copy number. Instead, it infers copy number from statistical deviations within a mixed maternal–placental DNA population [1,10]. Detection thresholds depend on fetal fraction, sequencing depth, bin configuration, GC correction, and noise modeling [11,12,17,18]. Accordingly, analytical “resolution” in NIPT is probabilistic rather than absolute. A CNV of a given size may be detectable under favorable conditions but undetectable in the presence of low fetal fraction or increased technical variability. Recognition of this probabilistic framework is essential for interpreting validation studies, comparing laboratory performance, and counseling patients regarding the inherent limitations of genome-wide CNV screening. The sequential pathway from placental cfDNA release to clinical interpretation, and the corresponding layers of uncertainty introduced at each stage, are summarized in Figure 1.

Schematic overview of the sequential analytical and biological steps involved in genome-wide copy number variant (CNV) detection by non-invasive prenatal testing (NIPT). Cell-free DNA (cfDNA) released predominantly from apoptotic placental syncytiotrophoblasts enters the maternal circulation as a mixed population of placental and maternal DNA fragments. Following plasma extraction, massively parallel sequencing is performed using shallow whole-genome sequencing (sWGS) or targeted sequencing approaches. Sequencing reads are aligned to the reference genome and aggregated into genomic bins for quantitative analysis.

Subsequent bioinformatic processing incorporates normalization procedures including GC-content correction, reference-based modeling, and noise reduction algorithms. Statistical deviations in read-depth representation are then interpreted as potential chromosomal or subchromosomal imbalances. At each stage of the process, distinct analytical and biological sources of uncertainty may influence signal interpretation, including low fetal fraction, confined placental mosaicism (CPM), maternal copy number variants (CNVs), maternal mosaicism, vanishing twin phenomenon, sequencing noise, and platform-dependent analytical thresholds.

The figure emphasizes that CNV-NIPT does not directly evaluate fetal genomic material but instead infers copy number changes probabilistically from a mixed maternal–placental cfDNA signal. Consequently, clinical interpretation reflects both analytical performance and underlying biological complexity.

Abbreviations: CNV, copy number variant; NIPT, non-invasive prenatal testing; cfDNA, cell-free DNA; sWGS, shallow whole-genome sequencing; CPM, confined placental mosaicism.

## 3. Biological Constraints

A central limitation of CNV detection by NIPT lies not only in analytical methodology but in the biological origin and composition of cfDNA. Because cfDNA analyzed in maternal plasma reflects a complex mixture of maternal and placental DNA, biological signal heterogeneity inherently constrains interpretive certainty. Unlike invasive diagnostic testing, which evaluates fetal cells directly, cfDNA screening infers fetal genomic status indirectly through a placental proxy. Consequently, biological discordance and mosaicism represent intrinsic, rather than exceptional, features of this testing modality.

### 3.1. Placental Origin of cfDNA

Fetal-derived cfDNA in maternal plasma originates predominantly from placental trophoblasts [1,8]. Following trophoblast apoptosis, placental DNA fragments are released into the maternal circulation and mixed with abundant maternal cfDNA [21]. Because placental and fetal tissues may undergo independent post-zygotic mitotic events, placental genomic constitution does not always reflect fetal genomic status. Consequently, CNVs detected by NIPT may represent placental-confined abnormalities rather than true fetal imbalances [9].

This placental origin represents a fundamental limitation of NIPT, as the assay evaluates placental rather than direct fetal genomic representation. Importantly, this biological uncertainty cannot be fully eliminated by increased sequencing depth or improved bioinformatic analysis.

### 3.2. Confined Placental Mosaicism (CPM)

Confined placental mosaicism (CPM) refers to chromosomal abnormalities present in placental tissue but absent in the fetus and occurs in approximately 1–2% of chorionic villus sampling cases [9,22]. Arising from post-zygotic mitotic errors, CPM may produce variable proportions of abnormal trophoblastic cells, directly influencing cfDNA signal intensity in maternal plasma [23].

Low-level placental mosaicism may generate weak or borderline CNV signals, whereas high-level mosaicism can produce apparently significant abnormalities despite a normal fetal karyotype. In addition, mosaicism may distort estimated CNV size because cfDNA analysis reflects aggregate placental copy number representation rather than direct fetal genomic architecture [23].

### 3.3. Maternal Genomic Contributions

Maternal genomic variation represents an additional source of biological complexity because most cfDNA in maternal plasma is maternal in origin. Maternal CNVs, constitutional mosaicism, and age-related clonal hematopoiesis may generate aberrant read-depth patterns that mimic fetal abnormalities [12,24]. In some cases, these signals may be misattributed to the fetus without confirmatory maternal testing.

Rarely, discordant or complex NIPT findings may also reflect occult maternal malignancy, as tumor-derived cfDNA can produce multiple aneuploid or CNV signals inconsistent with fetal viability [10,25]. These observations highlight that cfDNA analysis represents a systemic genomic signal rather than an exclusively fetal assay.

### 3.4. Vanishing Twin

Vanishing twin phenomenon represents another biological confounder. In multifetal conceptions where one embryo undergoes early demise, residual placental tissue may continue to release cfDNA into maternal circulation for weeks following fetal loss [26]. This residual cfDNA can persist sufficiently long to generate abnormal NIPT signals, including CNVs, unrelated to the surviving fetus. Because cfDNA clearance kinetics vary and the timing of embryonic demise may be clinically unrecognized, discordant CNV findings may reflect genomic contributions from a demised co-twin rather than ongoing fetal development [26,27]. This scenario illustrates the temporal dimension of biological complexity inherent in cfDNA interpretation.

### 3.5. Biological Signal Complexity Limits Interpretive Certainty

Collectively, placental origin, confined placental mosaicism, maternal genomic variation, occult malignancy, and vanishing twin phenomena illustrate that cfDNA is a composite biological signal shaped by multiple genomic contributors. These biological variables interact with fetal fraction and clonal architecture to modulate signal amplitude and detectability [26,27]. Accordingly, biological signal complexity imposes intrinsic limits on interpretive certainty in CNV-based NIPT. Even analytically robust platforms cannot fully resolve discordance arising from placental–fetal divergence or maternal genomic background. Recognition of these constraints is essential for accurate counseling, responsible reporting, and realistic appraisal of genome-wide CNV screening performance (Table 2) [20,21,22,23,24,25,26,27].

## 4. Clinical Validity of CNV Detection by NIPT

Clinical validity refers to the ability of a test to accurately and reliably detect clinically meaningful genomic imbalances in the intended population. In the context of genome-wide CNV detection by NIPT, clinical validity is influenced not only by analytical performance but also by population prevalence, CNV size distribution, biological mosaicism, and case ascertainment bias [1,2] (Table 3).

Early validation studies demonstrated that genome-wide CNV detection using shallow whole-genome sequencing is technically feasible, particularly for large CNVs in enriched or high-risk cohorts [18]. In these settings, reported sensitivities for CNVs >10 Mb frequently exceeded 80%, with specificity approaching 98% [18]. However, many of these studies relied on retrospectively selected abnormal cases, limiting direct applicability to routine screening populations.

Subsequent large-scale implementation studies produced more heterogeneous results. In cohorts exceeding 50,000 pregnancies, sensitivity for subchromosomal CNVs >7 Mb remained relatively high, but positive predictive value (PPV) varied substantially depending on CNV size, fetal fraction, and underlying population risk [17,28]. In predominantly general-risk populations, PPV declined considerably for smaller CNVs, often falling below 40% for CNVs ≥5 Mb [29]. These findings reinforce that predictive value is strongly influenced not only by analytical performance, but also by disease prevalence and referral patterns.

Targeted microdeletion screening has shown similar variability. SNP-based assays for recurrent microdeletion syndromes, including 22q11.2 deletion syndrome, demonstrated high analytical sensitivity in enriched validation cohorts [19]. However, real-world PPV in broader clinical populations proved substantially lower [20], highlighting the effects of cohort selection, ascertainment bias, and incomplete diagnostic follow-up.

Interpretation is further complicated by incomplete outcome data and methodological heterogeneity across studies. Many reports calculate PPV only among cases with available confirmatory testing, potentially introducing verification bias [28]. In addition, differences in laboratory algorithms, sequencing platforms, CNV size thresholds, and reporting criteria complicate cross-study comparisons [25,26,27,28,29,30,31]. Detection performance also declines for smaller or mosaic CNVs because reduced signal amplitude lowers both sensitivity and PPV [9].

Overall, published evidence supports reasonable performance for detection of large CNVs in enriched populations, whereas clinical validity remains considerably more variable in general screening settings. A comparative overview of major validation studies and reported performance metrics is summarized in Table 4.

Clinically, these findings support a cautious and context-dependent application of genome-wide CNV-NIPT. Use in enriched or contingent screening settings may provide clinically meaningful information, whereas first-tier application in general populations is associated with substantially lower predictive value. Accordingly, pre-test counseling should explicitly address the probabilistic nature of results and the likelihood of requiring confirmatory invasive testing following positive findings.

Therefore, while genome-wide CNV screening represents a technological advance, its clinical validity remains context-dependent. Transparent reporting of cohort composition, fetal fraction distribution, CNV size thresholds, and diagnostic follow-up rates is essential to accurately assess real-world performance and to support responsible clinical implementation. Published validation studies of genome-wide CNV detection by NIPT demonstrate substantial heterogeneity in study design, CNV size thresholds, and reported performance metrics. Key characteristics and reported performance outcomes of representative validation cohorts are summarized in Table 5 [17,18,19,20,28,29,30].

## 5. Clinical Interpretation and Reporting Challenges

The expansion of NIPT from whole-chromosome aneuploidy screening to genome-wide CNV detection has introduced substantial interpretive complexity. Unlike common trisomies, for which large validation datasets and well-characterized phenotypic spectra exist, subchromosomal CNVs encompass a broad range of genomic sizes, gene contents, penetrance profiles, and clinical expressivity. Consequently, interpretation and reporting frameworks for CNV-based NIPT remain less standardized and more variable across laboratories.

A central challenge lies in distinguishing screening from diagnostic paradigms. Professional societies have emphasized that NIPT is a screening test and that positive results require confirmatory invasive diagnostic testing [4,16]. However, as genome-wide CNV panels increase in scope and complexity, patients may perceive highly detailed genomic reports as approaching diagnostic certainty, despite NIPT remaining a screening test that requires confirmatory invasive diagnostic testing following positive findings. This perception may be reinforced when laboratory reports include detailed gene content, potentially exceeding the evidentiary basis of a probabilistic screening assay.

### 5.1. CNV Size Thresholds and Reporting Criteria

Laboratories differ in the minimum CNV size considered reportable, with commonly applied genome-wide thresholds ranging from 5 to 10 Mb [17,28]. Smaller recurrent microdeletions may be reported under targeted panel frameworks, while other subchromosomal imbalances below predefined size thresholds are typically suppressed. These thresholds reflect analytical performance and validation constraints rather than biological or clinical boundaries.

The absence of harmonized reporting standards complicates inter-laboratory comparison and may lead to variable clinical management for similar genomic findings. Furthermore, the clinical significance of intermediate-size CNVs (e.g., 5–10 Mb) may depend heavily on gene content and dosage sensitivity, raising questions about consistency in reporting practices.

### 5.2. Variants of Uncertain Significance (VUSs)

In diagnostic chromosomal microarray analysis, interpretation frameworks for CNVs are guided by established classification systems and curated databases. In contrast, NIPT-based CNV detection often lacks sufficient resolution or breakpoint precision to allow definitive classification. As a result, laboratories may avoid reporting variants of uncertain significance (VUSs), whereas others may report select findings deemed “likely pathogenic.”

The decision not to report VUSs may reduce patient anxiety but risks under-communicating genomic uncertainty. Conversely, reporting uncertain findings may generate psychological distress and increase invasive diagnostic procedures without clear benefit. The balance between transparency and clinical utility remains unresolved [27].

### 5.3. Mosaic and Borderline Calls

Mosaic CNVs represent a particular interpretive challenge. Reduced signal amplitude may reflect low fetal fraction, confined placental mosaicism, maternal mosaicism, or technical noise [9]. Borderline z-scores near reporting thresholds create ambiguity regarding whether a deviation represents true biological mosaicism or stochastic variation.

Some laboratories include qualitative comments regarding suspected mosaicism, while others apply binary reporting frameworks. The lack of quantitative mosaic fraction estimates further limits clinical interpretation and counseling precision.

### 5.4. Incidental and Maternal Findings

Genome-wide NIPT has revealed incidental maternal genomic abnormalities, including large CNVs and occult malignancies [10,25]. These findings raise ethical and clinical questions regarding disclosure, follow-up responsibility, and maternal autonomy.

Unlike fetal findings, maternal genomic abnormalities may have direct health implications for the pregnant individual. However, the primary intent of NIPT is fetal screening, and policies governing disclosure of maternal findings vary considerably. Clear pre-test counseling regarding the potential for incidental maternal findings is increasingly recognized as essential.

### 5.5. Counseling and Risk Communication

PPV for CNV detection is highly dependent on population prevalence and CNV size [17,29]. Yet PPV is often misunderstood in clinical counseling. Patients may interpret a “positive” result as diagnostic, despite its probabilistic nature.

Effective counseling requires transparent communication of uncertainty, including the possibility of false positives due to placental mosaicism, maternal CNVs, or technical artifacts. Genetic counseling resources may be strained as genome-wide panels increase the volume and complexity of findings. In clinical practice, it is essential to emphasize that genome-wide CNV-NIPT does not provide diagnostic certainty and should not be used to guide irreversible clinical decisions without confirmatory testing.

### 5.6. Toward Harmonized Reporting Frameworks

**Figure 2:**
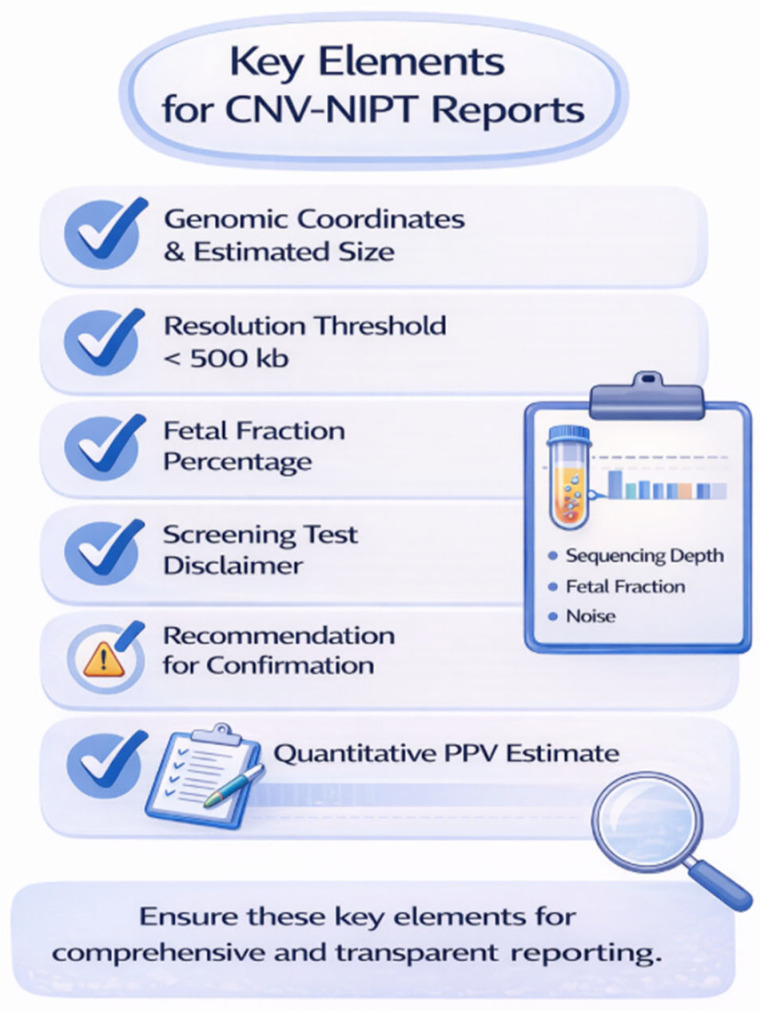
Key elements recommended for reporting and clinical interpretation of CNV-based non-invasive prenatal testing results.

Given the substantial interpretive variability surrounding genome-wide CNV detection by NIPT, the development of harmonized reporting standards is warranted. Standardization would enhance transparency, facilitate inter-laboratory comparability, and support consistent clinical counseling. A proposed minimum reporting framework is outlined in Table 6 [4,6,16,17,23,27,28,29,30,31,32] and summarized in Figure 2.

Schematic representation of the principal analytical and clinical elements recommended for standardized reporting of genome-wide CNV detection by NIPT. The figure highlights the central role of transparent reporting in supporting appropriate clinical interpretation, genetic counseling, and follow-up decision-making.

Recommended report components include: (1) genomic coordinates of the detected CNV together with the reference genome build; (2) estimated CNV size and chromosomal location; (3) fetal fraction at the time of analysis; (4) assay-specific analytical limitations, including validated resolution thresholds; (5) qualitative assessment of possible mosaicism when applicable; (6) estimated positive predictive value (PPV) or contextual risk interpretation when available; and (7) a clear statement emphasizing that NIPT is a screening rather than diagnostic test.

The figure also emphasizes the importance of recommending confirmatory invasive diagnostic testing following positive or uncertain findings, preferably by amniocentesis in situations where placental mosaicism may confound chorionic villus sampling (CVS) interpretation. Standardized reporting practices may improve inter-laboratory consistency, facilitate comparison across studies, and reduce the risk of overinterpretation of probabilistic screening data.

Abbreviations: CNV, copy number variant; NIPT, non-invasive prenatal testing; PPV, positive predictive value; CVS, chorionic villus sampling.

Adoption of such standardized reporting elements would align analytical capability with responsible clinical interpretation and help mitigate the risk of overextension of screening data beyond validated performance parameters.

Ultimately, clinical interpretation challenges reflect the convergence of biological complexity, probabilistic analytics, and variable evidentiary bases. As technological capabilities expand, careful alignment of reporting practices with validated performance data will be critical to preserving clinical utility and patient trust.

## 6. Discussion

Genome-wide CNV detection by NIPT extends aneuploidy screening into an area traditionally occupied by diagnostic chromosomal microarray analysis. However, despite increasing analytical sophistication, interpretation remains constrained by the biological complexity of placentally derived cfDNA. CNV-NIPT therefore remains a probabilistic screening approach rather than a direct assessment of the fetal genome.

Current evidence demonstrates reasonable detection performance for large CNVs, particularly in enriched or high-risk populations [17,18]. However, performance declines for smaller CNVs and in lower-risk cohorts, where positive predictive value becomes strongly prevalence-dependent [29]. Biological factors including confined placental mosaicism, maternal CNVs, and mosaic hematopoiesis further limit interpretive certainty [24,30].

Future improvements may depend less on sequencing depth alone and more on improved biological discrimination of cfDNA sources. Emerging approaches including methylation profiling, fragmentomics, and cfRNA integration may enhance tissue-of-origin characterization and reduce misclassification of placental or maternal signals [6,12,32]. At the same time, increasing analytical sensitivity must be balanced against the risk of detecting clinically ambiguous or low-level mosaic findings [9].

Standardization remains an important unmet need. Heterogeneity in CNV size thresholds, reporting practices, and follow-up completeness complicates comparison across laboratories and studies [28]. Consensus frameworks for genome-wide CNV-NIPT reporting and interpretation, together with clear pre-test counseling and confirmatory diagnostic testing pathways, will be essential for responsible clinical implementation [4,16].

**Figure 3:**
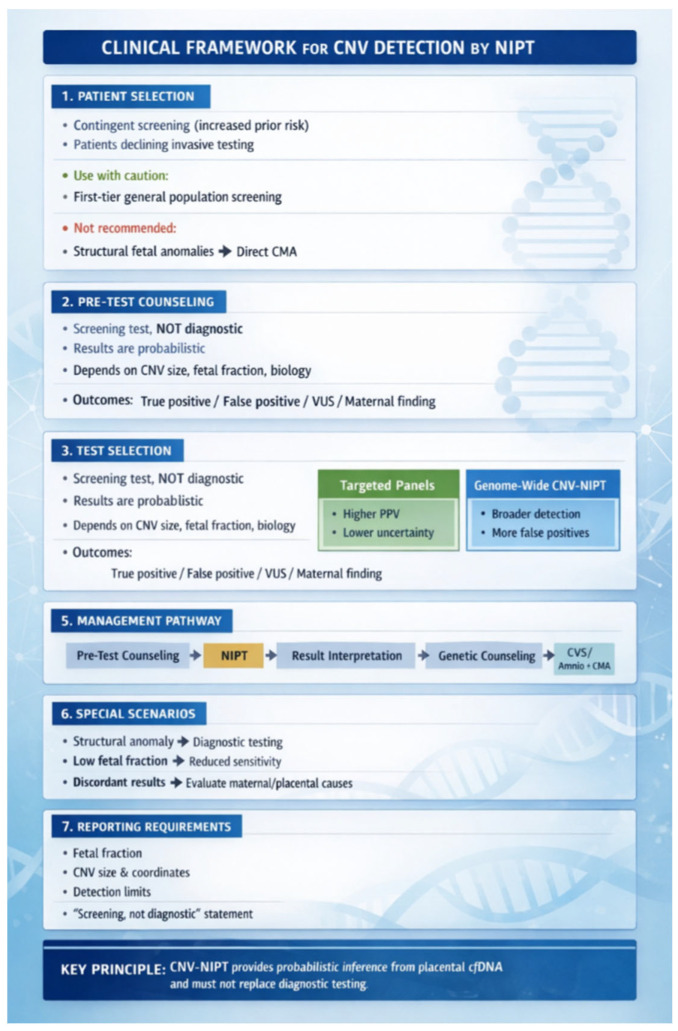
Proposed clinical framework for genome-wide CNV detection by non-invasive prenatal testing (NIPT).

Ultimately, the future clinical utility of genome-wide CNV-NIPT will depend on integration of improved biological insight, harmonized validation standards, and transparent communication of uncertainty. A proposed clinical framework integrating these analytical, biological, and clinical considerations is summarized in Figure 3.

Compared with alternative prenatal genomic approaches, genome-wide CNV-NIPT offers the major advantage of non-invasive risk assessment, but it remains fundamentally distinct from diagnostic technologies such as chromosomal microarray analysis (CMA). Invasive diagnostic workflows using amniocentesis or chorionic villus sampling combined with CMA continue to provide higher diagnostic resolution and direct fetal genomic characterization [6,7]. Recent studies have also highlighted the increasing role of exome sequencing and genome sequencing in the evaluation of structurally abnormal fetuses, particularly when conventional cytogenetic testing is nondiagnostic [33,34]. Emerging high-depth sequencing strategies and fetal genome reconstruction approaches may further improve analytical sensitivity for smaller CNVs and de novo variants, although these methods remain computationally intensive and clinically investigational [27,35]. In parallel, long-read sequencing technologies have shown promise for resolving structurally complex genomic regions, repetitive elements, and balanced rearrangements that are difficult to characterize using conventional short-read cfDNA analysis [36]. Nevertheless, even advanced sequencing approaches remain constrained by the biological limitations of placentally derived cfDNA, including confined placental mosaicism and maternal genomic background effects [9,24]. Consequently, although genome-wide CNV-NIPT may increasingly complement prenatal genomic evaluation, invasive diagnostic testing remains essential when definitive fetal genomic diagnosis is required.

An additional limitation of the current evidence base is that many validation studies were performed in enriched or referral populations with increased prior risk of chromosomal abnormalities, potentially inflating reported positive predictive values and limiting generalizability to routine screening settings [17,28,29]. Furthermore, substantial variability exists among sequencing platforms, fetal fraction thresholds, bioinformatic pipelines, and reporting criteria, complicating direct comparison of performance metrics across studies [25,26,27,28,29,30]. Many cohorts also suffer from incomplete confirmatory follow-up, selective diagnostic verification, and loss to follow-up after pregnancy termination, all of which may introduce verification bias and distort estimates of true clinical performance. Consequently, reported sensitivity and PPVs should be interpreted cautiously, particularly when extrapolating validation data to lower-risk populations or across different laboratory methodologies.

The proposed clinical workflow integrates patient selection, pre-test counseling, test performance characteristics, interpretation, and follow-up strategies for genome-wide CNV detection by NIPT. The framework emphasizes that CNV-NIPT should be regarded as a probabilistic screening modality based on placental cfDNA analysis rather than direct fetal genomic assessment.

The pathway begins with clinical indication assessment and pre-test counseling, including discussion of test limitations, false-positive and false-negative risk, potential incidental findings, and the possible need for confirmatory invasive testing. Following sequencing and bioinformatic analysis, interpretation of CNV findings is influenced by fetal fraction, CNV size, placental mosaicism, maternal genomic background, and assay-specific analytical thresholds.

Negative results should be interpreted within the context of assay resolution limits and residual risk, whereas positive or uncertain findings require confirmatory diagnostic testing, preferably by amniocentesis when placental mosaicism is suspected. The framework also incorporates post-test genetic counseling and consideration of chromosomal microarray analysis (CMA) for definitive genomic characterization.

Overall, the figure illustrates the interaction between analytical validity, biological complexity, and clinical decision-making, emphasizing that responsible implementation of genome-wide CNV-NIPT requires transparent communication of uncertainty and adherence to evidence-based diagnostic pathways.

Abbreviations: CNV, copy number variant; NIPT, non-invasive prenatal testing; cfDNA, cell-free DNA; CMA, chromosomal microarray analysis; PPV, positive predictive value.

From an implementation standpoint, genome-wide CNV detection by NIPT should be integrated cautiously into prenatal care pathways. Current evidence supports its use primarily as a secondary or contingent screening tool rather than as a universal first-tier test. In particular, its application in low-risk populations should be balanced against reduced positive predictive value, increased detection of uncertain findings, and the potential for unnecessary invasive procedures. Alignment with existing professional guidance from American College of Medical Genetics and Genomics and International Society for Prenatal Diagnosis remains essential to ensure appropriate clinical use.

## 7. Conclusions

Genome-wide copy number variant detection by non-invasive prenatal testing represents a significant technological evolution in prenatal screening; however, its clinical utility remains intrinsically constrained by biological signal complexity and probabilistic analytical resolution. While detection of large subchromosomal imbalances is increasingly robust, performance varies with CNV size, fetal fraction, population prevalence, and placental mosaicism. The placental origin of cfDNA fundamentally limits interpretive certainty, reinforcing the distinction between screening inference and diagnostic confirmation. As genome-wide panels expand into lower-risk populations, careful alignment of analytical capability with validated clinical performance is essential to avoid overinterpretation and unintended harm. Future progress will depend not solely on increased sequencing depth, but on improved biological discrimination of cfDNA sources, harmonized reporting standards, transparent validation data, and rigorous outcome studies. At present, genome-wide CNV detection by NIPT should be considered a complementary screening approach rather than a replacement for diagnostic testing, with its clinical use guided by population risk, pre-test counseling, and adherence to established professional recommendations.

## Figures and Tables

**Table 1:** Comparison of principal sequencing and analytical approaches used for CNV detection by NIPT.

Approach	Typical Sequencing Strategy	Genomic Coverage	Typical CNV Detection Range	Main Advantages	Main Limitations
Shallow Whole-Genome Sequencing (sWGS)	Low-coverage whole-genome massively parallel sequencing (typically ~0.1–0.5× coverage)	Genome-wide	Typically >5–10 Mb (platform-dependent)	Broad genomic coverage; capable of detecting genome-wide CNVs and aneuploidies; scalable for population screening	Lower resolution for small CNVs; strongly dependent on fetal fraction and sequencing depth; susceptible to noise and GC bias
Targeted Sequencing Panels	Enrichment of predefined genomic regions associated with recurrent microdeletion/microduplication syndromes	Limited to selected loci	Small recurrent CNVs at targeted loci (e.g., 22q11.2 deletion)	Higher effective read depth at selected regions; improved sensitivity for targeted syndromes	No genome-wide detection capability; locus-specific bias; limited flexibility
SNP-Based Methods	Analysis of polymorphic SNP patterns combined with targeted sequencing and statistical modeling	Selected chromosomal and subchromosomal regions	Typically targeted recurrent CNVs and selected aneuploidies	Improved discrimination of maternal and fetal haplotypes; useful for triploidy and some mosaic cases	Limited genomic scope; dependent on informative SNP distribution; complex bioinformatic interpretation
Deep Whole-Genome Sequencing	Higher-coverage genome-wide sequencing compared with standard sWGS	Genome-wide	Potentially <5 Mb under optimized conditions	Improved signal-to-noise ratio and theoretical resolution	Increased sequencing cost; higher computational burden; biological limitations remain unresolved
Fragmentomic/Multi-omic Approaches (Emerging)	Integration of fragment size analysis, methylation profiling, or cfRNA signatures with cfDNA sequencing	Genome-wide with tissue-specific inference	Investigational	Potential improvement in tissue-of-origin discrimination and biological specificity	Experimental; limited clinical validation; lack of standardized implementation

Abbreviations: CNV, copy number variant; NIPT, non-invasive prenatal testing; SNP, single-nucleotide polymorphism; cfDNA, cell-free DNA; cfRNA, cell-free RNA; sWGS, shallow Whole-Genome Sequencing.

**Table 2:** Sources of Discordant CNV-NIPT Results and Their Mechanistic Basis.

Source of Discordance	Biological Mechanism	Expected Signal Pattern	Clinical Consequence
Confined placental mosaicism	Post-zygotic trophoblast error	Reduced amplitude CNV	False positive
Maternal CNV	Constitutional maternal variant	Full amplitude but maternal origin	False positive
Maternal mosaicism	Clonal hematopoiesis	Low-level deviation	Ambiguous call
Vanishing twin	Residual trophoblast DNA	Transient CNV signal	Discordant result
Low fetal fraction	Signal dilution	False negative risk	Reduced sensitivity

Abbreviations: CNV, copy number variant.

**Table 3:** Summary of published CNV-NIPT validation studies.

Study	Study Design	Sample Size	CNV Threshold (Reportable Range)	Sensitivity	Specificity	PPV	Population Type
[18]	Retrospective performance evaluation using low-coverage WGS (FCAPS), compared with karyotype/microarray	919 pregnancies (archived/selected cohort)	CNVs 1–129 Mb; subgrouped >10 Mb vs. <10 Mb	84.21% overall	98.42% overall	NR (authors note PPV not assessable due to selected cohort)	High-risk/selected (had invasive confirmation; not a general screening cohort)
[17]	Retrospective clinical cohort (“real-world” genome-wide cfDNA) with performance calculated in subset with diagnostic outcomes	55,517 submitted (53,099 reportable); performance subset n = 1569 with diagnostic outcomes	Subchromosomal CNVs ≥7 Mb (plus select microdeletions <7 Mb)	94.1% (subchromosomal CNVs)	96.7% (subchromosomal CNVs)	72.6% (subchromosomal CNVs)	Mixed-risk clinical population (increasing proportion with no high-risk indication over time)
[28]	Retrospective analysis of screen-positive CNV cases from large genome-wide cfDNA program (focus on PPV among those with outcomes)	86,902 screened; 490 CNV-positive cases analyzed	Reportable CNV defined as ≥7 Mb	NR	NR	74.2% overall PPV among cases with diagnostic outcomes (244/490 had outcomes)	Largely general clinical screening (singleton pregnancies)
[29]	Retrospective first-tier genome-wide NIPT program with follow-up CMA for CNV cases	59,771 successfully tested	CNVs ≥5 Mb	63.6%	97.6%	33.9%	Predominantly general screening cohort (first-tier GW-NIPT)
[19]	Case–control validation of SNP-based cfDNA test for 22q11.2 deletion	400 (10 affected, 390 controls)	22q11.2 deletion (microdeletion-targeted; size depends on assay definition)	90%	99.74%	NR (case–control design; PPV not generalizable)	Enriched/validation set (not population screening)
[20]	Retrospective clinical experience of SNP-based screening for 22q11.2 deletion	21,948 submitted	22q11.2 deletion (targeted microdeletion screening)	NR			
[30]	Technical/clinical validation of low-pass WGS cfDNA CNV calling vs. CMA	34 pregnancies (40 CNVs observed; 32 evaluable)	Reported by size strata: <3 Mb vs. ≥3 Mb	90.6% overall (100% for ≥3 Mb; 78.6% for <3 Mb)	NR	NR	High-risk (ultrasound findings/family history; invasive testing)

Abbreviations: CNV, copy number variant; NIPT, non-invasive prenatal testing; PPV, positive predictive value; NR, not reported/not estimable from published data; SNP, single-nucleotide polymorphism; cfDNA, cell-free DNA; WGS, Whole-Genome Se-quencing.

**Table 4:** Sources of heterogeneity in CNV-NIPT studies: why results are not directly comparable.

Domain of Heterogeneity	Study-Level Variation	Effect on Reported Performance	Direction of Bias	Example Evidence
Cohort selection	Enriched/high-risk vs. general screening populations	Alters prevalence of CNVs and case mix	Enriched cohorts inflate sensitivity and PPV; screening cohorts reduce PPV	[18,29]
Clinical indication	Ultrasound anomalies, prior risk vs. unselected population	Higher pre-test probability increases PPV	Overestimation of real-world performance in selected cohorts	[17,31]
Follow-up completeness	Full diagnostic confirmation vs. partial or selective follow-up	Affects accuracy of PPV and false-positive rates	Incomplete follow-up may inflate PPV or obscure false positives	[17,28]
Outcome ascertainment	Inclusion of only confirmed cases vs. all screen-positive cases	Introduces verification bias	Overrepresentation of true positives	[28]
CNV size thresholds	Variable reporting cutoffs (e.g., ≥5 Mb vs. ≥7 Mb vs. ≥10 Mb)	Smaller CNVs reduce detection performance and PPV	Lower thresholds increase false positives and variability	[17,29]
Sequencing platform	Shallow WGS vs. targeted SNP-based approaches	Affects resolution, sensitivity, and genomic coverage	Platform-dependent variability in detection rates	[19,20]
Bioinformatic pipelines	Different normalization, binning, and calling algorithms	Influences signal detection and noise handling	Variable sensitivity/specificity across laboratories	[17,18]
Fetal fraction distribution	Variation across cohorts (e.g., gestational age, BMI)	Directly affects signal amplitude and detectability	Lower fetal fraction reduces sensitivity	[18,29]
Mosaicism handling	Inclusion/exclusion or inconsistent reporting of mosaic CNVs	Affects both sensitivity and PPV estimates	Mosaic cases may be misclassified or excluded	[9,17]
Reporting practices	Differences in VUS reporting and size thresholds	Alters apparent detection rates and clinical relevance	Under- or over-reporting of clinically uncertain findings	[20,27]

Abbreviations: BMI, body mass index; CNV, copy number variant; PPV, positive predictive value; SNP, single-nucleotide polymorphism.

**Table 5:** Clinical validity of CNV detection by NIPT across different screening contexts.

Screening Context	Population Type	CNV Scope	Detection Rate (Sensitivity)	PPV	Key Observations	Clinical Implication
High-risk population	Ultrasound anomalies/invasive testing cohort	Genome-wide	High (>80–90% for CNVs >7–10 Mb) [18,30]	High (~70–80%) [18]	Enriched cohorts; retrospective validation designs [18,30]	May overestimate performance in screening settings
General population	Unselected/clinical screening	Genome-wide	Moderate; size-dependent [17,29]	Lower (~30–50%) [28,29]	Lower prevalence reduces PPV; incomplete follow-up common [28,29]	Increased false positives and counseling burden
First-tier screening	Broad population without prior risk stratification	Genome-wide	Variable; reduced for smaller CNVs [29]	Low–moderate (~30–40%) [29]	Applied universally; low prior probability reduces predictive value [29]	Limited clinical utility for small CNVs
Contingent screening	Intermediate/high-risk after prior screening	Genome-wide	Higher than first-tier [17,28]	Moderate–high [17,28]	Risk enrichment improves performance characteristics [17]	More consistent with guideline-based implementation
Targeted panels	Mixed-risk populations	Selected CNVs (e.g., 22q11.2)	High for specific loci [19]	Variable; lower in real-world than validation cohorts [20]	Case–control validation inflates performance estimates [19,20]	Limited scope but clearer interpretation
Genome-wide vs. targeted	Mixed	Genome-wide vs. targeted	Genome-wide: broader but variable sensitivity [17,29]; Targeted: higher locus-specific sensitivity [19]	Genome-wide: variable PPV [29]; Targeted: often higher for specific CNVs [20]	Trade-off between breadth and reliability [17,20,29]	Genome-wide increases detection but also uncertainty

Abbreviations: CNV, copy number variant; NIPT, non-invasive prenatal testing; PPV, positive predictive value.

**Table 6:** Reporting and Clinical Interpretation of CNV-NIPT Results.

Domain	Recommended Reporting Element	Rationale	Clinical Implication
Analytical	Sequencing depth	Influences detection threshold	Context for resolution limits
Analytical	Fetal fraction (%)	Determines signal amplitude	Interpretation of negative results
CNV characteristics	Genomic coordinates (hg build)	Ensures reproducibility	Enables confirmatory testing
CNV characteristics	Estimated size (Mb)	Performance correlates with size	Smaller CNVs → lower PPV
Mosaicism	Qualitative or quantitative estimate	CPM confounds interpretation	May explain discordance
Interpretation	Estimated PPV (if available)	Prevalence-dependent	Informs counseling
Reporting statement	Explicit “screening, not diagnostic” disclaimer	Prevents overinterpretation	Supports ethical practice
Follow-up	Recommendation for invasive confirmation	Gold standard remains diagnostic	Avoids clinical mismanagement

Abbreviations: CNV, copy number variant; NIPT, non-invasive prenatal testing; PPV, positive predictive value.

## Data Availability

Not applicable.

## Abbreviations

The following abbreviations are used in this manuscript:

cfDNA	cell-free DNA
cfRNA	cell-free RNA
CMA	Chromosomal microarray analysis
CNV	Copy number variant
CPM	Confined placental mosaicism
GC	Guanine-cytosine
Mb	Megabase
NIPT	Non-invasive prenatal testing
PPV	Positive predictive value
SNP	Single-nucleotide polymorphism
sWGS	Shallow whole genome sequencing
VUS	Variant of uncertain significance
WGS	Whole-genome sequencing
